# Oral Perceptions of Fat and Taste Stimuli Are Modulated by Affect and Mood Induction

**DOI:** 10.1371/journal.pone.0065006

**Published:** 2013-06-05

**Authors:** Petra Platte, Cornelia Herbert, Paul Pauli, Paul A. S. Breslin

**Affiliations:** 1 Department of Psychology, University of Würzburg, Würzburg, Germany; 2 Institute of Psychology, German Sport University Cologne, Cologne, Germany; 3 Department of Nutritional Sciences, Rutgers University, New Brunswick, New Jersey, United States of America; 4 Monell Chemical Senses Center, Philadelphia, Pennsylvania, United States of America; German Institute of Human Nutrition Potsdam-Rehbruecke, Germany

## Abstract

This study examined the impact of three clinical psychological variables (non-pathological levels of depression and anxiety, as well as experimentally manipulated mood) on fat and taste perception in healthy subjects. After a baseline orosensory evaluation, ‘sad’, ‘happy’ and ‘neutral’ video clips were presented to induce corresponding moods in eighty participants. Following mood manipulation, subjects rated five different oral stimuli, appearing sweet, umami, sour, bitter, fatty, which were delivered at five different concentrations each. Depression levels were assessed with Beck’s Depression Inventory (BDI) and anxiety levels were assessed via the Spielberger’s STAI-trait and state questionnaire. Overall, subjects were able to track the concentrations of the stimuli correctly, yet depression level affected taste ratings. First, depression scores were positively correlated with sucrose ratings. Second, subjects with depression scores above the sample median rated sucrose and quinine as more intense after mood induction (positive, negative and neutral). Third and most important, the group with enhanced depression scores did not rate low and high fat stimuli differently after positive or negative mood induction, whereas, during baseline or during the non-emotional neutral condition they rated the fat intensity as increasing with concentration. Consistent with others’ prior observations we also found that sweet and bitter stimuli at baseline were rated as more intense by participants with higher anxiety scores and that after positive and negative mood induction, citric acid was rated as stronger tasting compared to baseline. The observation that subjects with mild subclinical depression rated low and high fat stimuli similarly when in positive or negative mood is novel and likely has potential implications for unhealthy eating patterns. This deficit may foster unconscious eating of fatty foods in sub-clinical mildly depressed populations.

## Introduction

The human gustatory system varies within subjects in its responsiveness to stimulation as a function of several biologically relevant variables including: time of day [1], hunger and nutritional state [2], eating habits [3], age [4], hormonal status [5], pregnancy [6], and neurotransmitter/organic psychological disorders [7], [8]. Individual differences in taste thresholds exist and are suspected to influence daily food intake and consequently body weight – although concrete evidence of this is still missing [9]. The principal taste qualities that are recognized by humans are sweet, sour, bitter, salty and umami (or savory). Until recently fat was not regarded as a taste stimulus. But recent studies suggest that fatty acids stimulate taste receptor cells and humans with genetic variants in their fatty acid transporter CD36 differ in their ability to detect fatty acids [10]–[13].

Studies about psychological influences on taste perception indicate that the taste system is sensitive to emotional and stressful manipulations. Ileri-Gurel et al [14]reported a significant decrease in glucose and salt thresholds after exposing healthy subjects to a stress test. Patients with major clinical depression have elevated thresholds for sugars (were less sensitive) [15], [16]. Interestingly, thresholds in these patients return to pre-depression levels after clinical recovery. Whereas it is unclear why depression affects taste thresholds, it may alter the neural pharmacology of taste or change the cognitive decision biases that results in higher threshold outcomes (or both) [17].

Addressing the neuropharmacological theory, Heath et al. [7] studied taste thresholds in healthy subjects before and after administration of a single dose of a serotonin (5-HT-specific) reuptake inhibitor, a noradrenaline (NA) reuptake inhibitor, or a placebo. Interestingly, the increase in synaptic 5-HT significantly reduced the taste thresholds for sucrose and quinine (increased sensitivity to them), and the increase in synaptic NA decreased quinine and citric acid thresholds (increased sensitivity). This is consistent with observations of depression associated with increased taste thresholds. These findings provide further evidence of the neuropharmacological plasticity of the human taste system. Since the serotonergic and the noradrenergic systems are involved in clinical anxiety and depression and are the main targets of antidepressants, changes in these systems may explain taste alterations in these patients.

In addition, results from animal studies suggest that rats decrease the number of times they initiate bouts of licking for NaCl and sucrose after administration of the selective serotonin reuptake inhibitor Paroxetine [18].

Here we examined interindividual differences in affect and mood dependent plasticity of the fat and taste perception systems. Specifically, we tested whether non-pathological variations in depression and anxiety assessed with validated clinical tools or experimental manipulations of mood via emotional video clips modulate oral fat and taste perception in healthy subjects. The major outcome variables were the rated oral intensities of the fat and taste stimuli on a general labeled magnitude scale.

## Methods

### Ethical Statement

The research protocol and informed consent forms were approved by the Institutional Review Board of Würzburg University.

### Subjects

Forty eight women and thirty two men aged 19 to 47 (body mass index [BMI; mass/height^2^]: 17,5 to 29,71)were recruited via posters and word of mouth in the city of Würzburg (mean (± SD) age 24±5 years) (see Table 1). *A priori* exclusion criteria were the presence of any acute or chronic disease, use of any prescription medication, a history of a clinical eating disorder, food allergies, and smoking more than 5 cigarettes per day.

**Table 1:** Sample characteristics (mean ± SD).

	All subjects (N = 80)	Men (n = 32)	Women (n = 48)	T (95%)	p
Age	24.0±5.1	25.1±5.1	23.3±5.1	−1.26	ns
BMI	22.6±3.3	24.1±3.0	21.7±3.1	−3.82	<0.001
Tobacco (cigarettes/day)	0.4±1.1	0.6±1.2	0.2±0.9	−1.516	ns
BDI	2.3±3.2	2.7±3.5	2.1±3.0	−0.655	ns
STAI- state	40.1±8.3	39.6±8.3	40.4±8.3	0.600	ns
STAI- trait	37.8±8.8	39.2±9.0	37.0±8.6	−0.946	ns

After passing this first screening procedure, written, informed consent was provided by each subject who was paid for their participation. After the baseline measurement and experimental inductions participants completed the 21– item Beck Depression Inventory [19], the 40– item self-report measure of trait and state anxiety symptoms [20] and a short questionnaire about physical and lifestyle characteristics. Sample characteristics are shown in Table 1. Body mass index (BMI) was also assessed. BMI was significantly higher in men than women (*t* = 3,82; p<0.001). Participants were all Caucasian.

### Mood Induction

Participants were shown three clips from movies for mood induction (for review of this technique see Hewig et al., [21]). Sadness was induced by a clip from “The Champ” (in which a boxer is lying severely injured on a table, while his young son watches him die [22], duration = 2∶51 min). Happiness was induced by a clip from “An Officer and a Gentleman” (in which the male hero goes to the factory where his girlfriend works to reunite with her, duration = 2∶03 min, [23]). A clip from a documentary about the processing and usage of copper (duration = 2∶02 min) was shown as a non-mood inducing, neutral control condition. To maintain the desired mood throughout the taste testing session, the main title music of the video clip was played in the background. A baseline taste test without any induction of emotions preceded the experimental manipulation to familiarize participants with the testing procedure. The baseline measurement was used as a “warm-up” to help subjects to concentrate on perceived intensities [24]. The baseline measurement was not compared with the results of the following mood inductions.

### Taste and Fat Stimuli

Five different stimuli, prototypical elicitors of sweet, umami, sour, bitter and fatty sensations, were administered to subjects at five concentrations and water, in ascending order. All stimuli but the fat stimuli were prepared by a pharmacist. The taste stimuli were fabricated by Caesar & Lorenz GmbH, Hilden, Germany. The water for the aqueous samples was from Fagron GmbH, Barsbüttel, Germany. Sucrose (sweet) was presented at 50, 100, 150, 200, 250 mmol/L; glutamate monopotassium salt (MPG) (umami) was presented at 25, 50, 75, 100 and 125 mmol/L; citric acid (sour) was presented at 0.06, 0.2, 0.6, 2 and 6 mmol/L; quinine sulfate (bitter) was presented at 0.534, 1.69, 5.34, 16.9, 53.4 mmol/L, and milk fat (fatty) was presented as mixtures of 0.2% fat milk with heavy cream to produce 0.2, 2, 4, 6, 8, and 10% milk fat (w/v) dairy solutions. Subjects were trained in the use of a general Labeled Magnitude Scale (gLMS) following published standard procedures [25]–[27]. The intensity ratings on the scale range from 0 = “barely detectable” to 100 = “strongest imaginable”. The anchor “strongest imaginable” was described as the strongest imaginable sensation of any kind. The gLMS is a pseudo-logarithmic, ratio -quality scale that has been experimentally and quantitatively validated in a series of publications against magnitude estimation, which yields ratio quality data as well [25], [26], [28]. A gLMS avoids ceiling effects as it is anchored against the strongest imaginable sensation and, therefore, contains a portion of the spectrum that our subjects were unlikely to use. Most other VAS are subject to ceiling effects and, therefore, their data can only be considered ordinal. The perceived intensity of the taste and fat stimuli during mood induction is the dependent variable. The baseline test was conducted to help the subjects concentrate on the movies and the taste and fat stimuli.

Subjects arrived after having refrained from all food, drink, smoking, or the use of toothpaste for 2 hours prior to testing. They received verbal and written instruction (via Microsoft PowerPoint presentation on a beamer). They started with a baseline taste test. After the baseline the approximately 2 min video clips were projected (using the same beamer) onto a screen mounted on the wall of the test room.

The session was divided into blocks: (1) baseline taste test, (2) induction of mood by movie (positive, negative and neutral films, presented in two pre-determined orders: e.g., positive, neutral, negative or negative, neutral, positive) - counterbalanced across subjects. They were instructed to watch the movies carefully. After the movies and the sensory testing were completed, changes in positive affect (PA) and negative affect (NA) were assessed with the Positive and Negative Affect Scale (PANAS) [29] to determine if the desired mood induction was successful. Directly after, participants started the taste tests in a randomized order by type of compound. The 10 ml solutions were offered in 30 mL-polypropylene medicine cups and presented in ascending order of concentration on a numbered tray. There was a 10 min break between blocks. Stimuli were prepared every two days and refrigerated. On the day of testing, the stimuli were brought to room temperature (∼21°) by sitting on the lab counter for 2 hours. The fat stimuli were prepared on the day of testing and also served at room temperature. Participants were asked to swish the solution in their mouth for 5 sec, then to judge the sample for total intensity and to rate the magnitude on a general labeled magnitude scale (gLMS). After the rating they expectorated the solution. Between test samples they were instructed to rinse their mouth with water and eat two bites of a matzah cracker. The dairy fat stimuli have smell-, taste – and texture properties. Since participants did not wear nose clips, all three orosensory factors could have been involved in their ratings. They were instructed to rate the perceived fattiness by evaluating their mouthfeel. The study lasted 2,5 to 3 hours per person and was conducted by two examiners, who worked together.

### Statistical Analysis

Data were analyzed using SPSS for Windows, version 18.0. Participant’s group characteristics (age, BMI, tobacco, BDI, STAI-state, STAI-trait) were analyzed using independent measures t-tests with gender as the independent variable. In the baseline Analysis of Variance (ANOVA) “gender” was used as the between factor and “taste intensity“ was used as a within group factor. As a test of the overall stimulatory impact of increasing concentrations, ratings were assessed for linear or quadratic trends within stimulus category. If significant interactions between either linear or quadratic trends and group or mood were identified, then trends and interactions were tested within the different groups separately. Repeated measures ANOVA and Bonferroni corrections for multiple group comparisons were used to analyze the PANAS data as a confirmation of successful mood manipulation. A oneway ANOVA was used for baseline testing. Two-factor repeated measures ANOVAs were used for the within group factors “mood” (positive, negative and neutral) and “taste intensity” (water plus five concentrations). The median split was used to classify groups into no versus mild subclinical depression, and low versus mild subclinical trait anxiety. These group variables were used in the ANOVAs as a between subjects factor. Spearman’s statistics were used for correlation analyses. Data were presented as mean ± SD, if not otherwise indicated. The significance level was set to p<0.05 false positive rate.

## Results

Table 2 depicts the mean positive and negative affect scales from the PANAS after the mood induction. A one way repeated measures ANOVA revealed for Positive Affect (PA) a significant main effect of mood manipulation (F(2,78) = 40.1, p<0.001). Post hoc pairwise comparisons with Bonferroni corrections for multiple comparisons revealed significant differences in PA between the positive and neutral movie (Mean Difference = 3.26; p<0.001) and the negative and neutral movie (Mean Difference = −1.36; p = 0.043). For Negative Affect (NA) the main effect of mood manipulation was also significant (F(2,78) = 35.1, p<0.001). Post hoc pairwise comparisons with Bonferroni corrections did not show a significant difference between the positive and neutral movies (Mean Difference: −0.27; p = ns), but NA was significantly higher after the negative movie compared to the neutral movie (Mean Difference: 2.05; p<0.001).

**Table 2:** Mean (± SD) positive and negative affect after mood induction in 80 subjects.

Positive Affect(PANAS)	M ± SD		mean difference	p
Positive movie	25.78±5.64	positive vs. neutral	3.26	p<0.001
Neutral movie	22.52±5.51			
Negative movie	21.16±4.80	negative vs. neutral	−1.36	p = 0.043
Negative Affect (PANAS)				
Positive movie	11.00±2.34	positive vs. neutral	−0.27	p = ns
Neutral movie	11.27±2.25			
Negative movie	13.05±3.28	negative vs. neutral	2.05	p<0.001

Thus, the movies had their desired effects as positive or negative mood manipulation procedures.

The test-retest reliability coefficients between the baseline and the neutral condition were r = .513 (citric acid), p<0.001; r = .609 (sucrose), p<0.001; r = .625 (quinine), p<0.001; r = .648 (umami), p<0.001; r = .746 (fat), p<0.001 with correlation magnitudes above 0.50 interpreted as acceptable retest reliability.

### Main Effects of Concentration and Mood on Taste Ratings

As a group, the participants were able to rate the intensity of the different oral stimuli as increasing with concentration, i.e., all concentration effects followed linear trends (sucrose: F (1,79) = 358.7, p<0.001; glutamate: F (1,79) = 105.2, p<0.001; quinine: F (1,79) = 243.2, p<0.001; fat: F (1,79) = 16.2, p<0.001).

**Figure 1:**
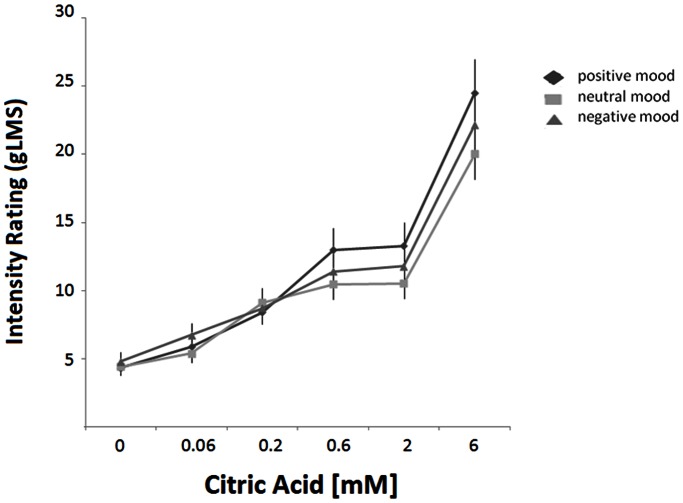
Mean intensity ratings of citric acid as a function of concentration after mood manipulation by positive, neutral, and negative video clips. Error bars are standard errors.

The induction of mood did not change the ratings of sucrose, glutamate, quinine and fat sensations (sucrose: F(2,78) = 0.19, p = ns; glutamate: F (2,78) = 0.16, p = ns; quinine: F (2,78) = 0.34, p = ns; fat: F (2,78) = 1.16, p = ns). Mood induction, however, changed the intensity ratings of citric acid (F (2,78) = 4.77, p = 0.011). Following both, the positive and negative mood manipulations, citric acid was rated as stronger tasting compared to after the neutral movie, especially at the higher concentrations (F (10,70) = 2.64; p = 0.004) (Figure 1).

Gender did not influence the taste ratings of sucrose (F (1,79) = 0.17; p = ns), glutamate (F(1,79) = 1.06; p = ns), citric acid (F(1,79) = 0.42; p = ns), quinine (F(1,79) = 3.08; p = ns), or dairy fat perception (F(1,79) = 0.18; p = ns). In addition, no significant interactions between gender and mood induction were found for sucrose (F(3,77) = 2.96; p = ns), glutamate (F(3,77) = 0.05; p = ns), citric acid (F(3,77) = 0.96; p = ns), quinine (F(3,77) = 0.89; p = ns), or fat(F(3,77) = 1.54; p = ns) ratings, so data from women and men were pooled.

### Effects of “Sub-clinical” Depression (BDI)

The group was divided by median split into no subclinical depression (0.30±0.465; n = 40) and mild subclinical depression (4.34±3.46; n = 40) subgroups. The median BDI score which divided the group was 2. These subgroups did not differ in their gender distribution (χ^2^ = 0.28; p = ns). Subjects from the mild subclinical depression group had significantly higher NA (negative affect) ratings throughout the study compared to the no depression group (for example NA after the neutral movie: 10.5±0.9 vs. 12.1±2.8, t = −3.58; p = 0.001). But the induction of negative mood was successful in the mild subclinical depression group as well, which is shown by their difference in NA between the neutral and the negative movie (12.1±2.8 vs. 14.1±3.7, T = −4.6; p<0.001). Not surprisingly, the correlation between STAI-trait and BDI was r = 0.69, p<0.001. Depression and anxiety are typically comorbid traits [30]. Because of the high correlation between those two concepts, we also tested within the group with mild subclinical depression for differences between those with no and mild subclinical anxiety [31].

#### Effect of “sub-clinical” depression on oral stimulus ratings

Baseline measurements revealed a significant difference in taste intensity ratings for sucrose between the groups with no and mild subclinical depression; people with mild subclinical BDI scores (tending toward greater depression) rated the taste of sucrose significantly higher (F (1,79) = 4.98, p = 0.028). For quinine intensity taste ratings, a significant interaction between BDI group and concentration was found (F (5,75) = 4.21, p = 0.001). The mild subclinical depression group compared to the no depression group rated quinine at high concentrations as stronger (p = 0.048). The level of anxiety in the group with mild subclinical depression did not have a significant impact on taste ratings. No differences in ratings were found between the groups with mild subclinical depression and no anxiety (n = 12) and mild subclinical depression and mild subclinical anxiety (n = 29) for sucrose (F (1,39) = 1.8, ns) or quinine (F (5,34) = 0.10, ns).

The groups with no and mild subclinical depression showed no significant differences in baseline measurements for glutamate, citric acid, or fat ratings (all p>0.1).

#### Interaction of “sub-clinical” depression and mood manipulation on oral stimulus ratings

**Figure 2:**
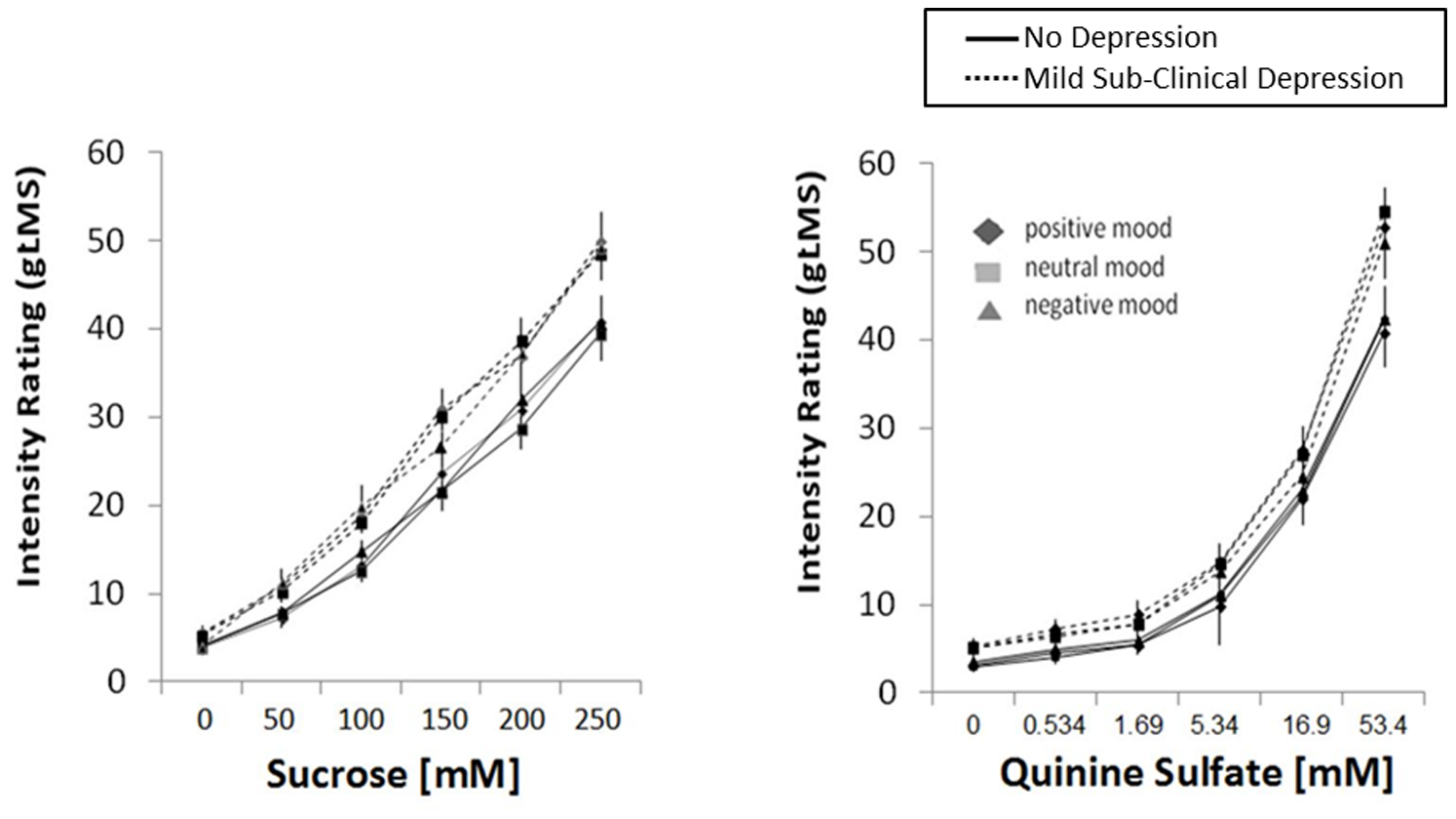
Mean intensity ratings (gLMS) of sucrose (left panel) and quinine sulfate (right panel) after positive, neutral, and negative mood induction by video clips in subjects with no depression (solid lines) and mild sub-clinical depression (dashed lines). Error bars are standard errors.

As indicated by significant between-subjects factor, the group with the mild subclinical depression rated sucrose (F(1,79) = 5.17; p = 0.026), quinine (F(1,79) = 5.78; p = 0.019) (Figure 2) and citric acid (F(1,79) = 4.20, p = 0.047) significantly higher compared to the no subclinical depression group after watching the mood-inducing movies regardless of its valence (positive and negative). Mild subclinical anxiety versus no anxiety within the group of mild subclinical depression had no effect on the ratings (all p>0.1).

**Figure 3:**
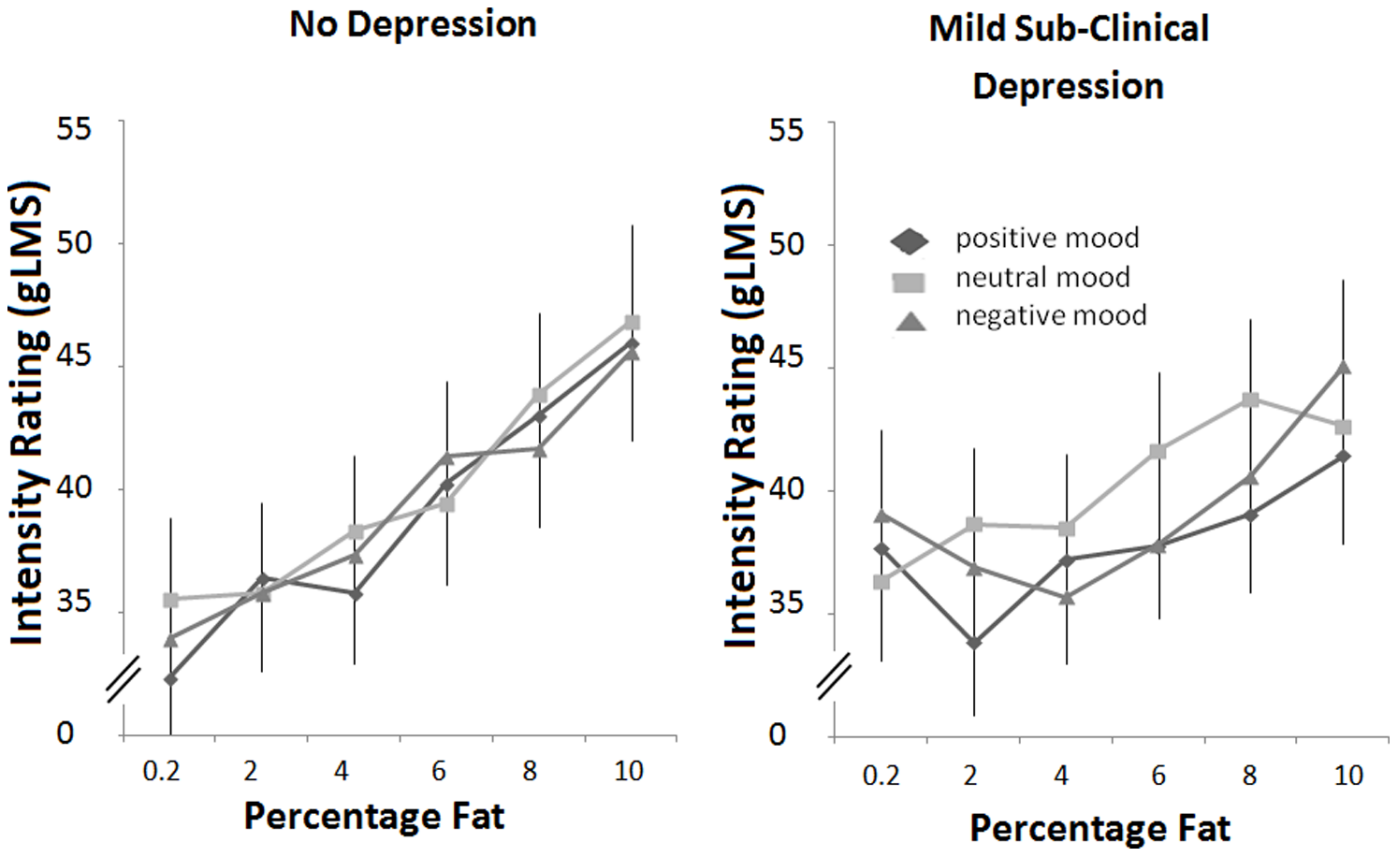
Mean intensity ratings (gLMS) of dairy fat stimuli after positive, neutral, and negative mood induction by video clips in subjects with no depression (left panel) and mild sub-clinical depression (right panel). Error bars are standard errors.

Importantly, the no depression and the mild subclinical depression groups differed regarding their fat intensity ratings of the ascending milk-cream mixtures depending on the video clip manipulations (Figure 3). Trend analysis for the three way interaction of mood x intensity × BDI group was significant for the quadratic trend (describing a response pattern that is U-shaped: higher ratings for lower concentrations) (F(1,79) = 8.14; p = 0.006), but not for the linear trend (F (1,79) = 0.11; p = ns). Further tests within groups revealed a significant mood × intensity interaction for a quadratic trend in the mild subclinical depression group (F(1,39) = 4.9; p = 0.03); this observation is underscored by the quadratic trends in ratings after positive (F 1,39) = 3.9; p = 0.05) and negative mood (F(1,39) = 9.9; p = 0.003) induction, but a linear trend after the neutral film (F 1,39) = 6.1; p = 0.018). In contrast, the intensity effects within the no depression group were linear after all three mood conditions. In other words, the mildly subclinical depressed subjects rated the fat stimuli as a linear function of concentration after the neutral film, but not after the induction of positive or negative mood. In essence, the mildly subclinical depression fatty ratings were flat as a function of fat concentration after mood induction, despite being presented the fat concentrations in ascending order.

### Effects of “Sub-clinical” Trait-anxiety (STAI)

The group was divided on the basis of their STAI score and median split into no (31.3±4.3; n = 43) and mild subclinical anxiety (45.3±6.4; n = 37) subgroups. The median STAI - trait score that was used to split the group was 37. The trait anxiety subgroups did not differ in their gender distribution (χ^2^ = 0.72, p = ns). Because of the high correlation between depression and anxiety, we tested within the mild subclinical anxiety subgroup for differences between those with no versus mild subclinical depression [20].

#### Effect of “subclinical” trait anxiety on perceived baseline ratings of oral stimuli

**Figure 4:**
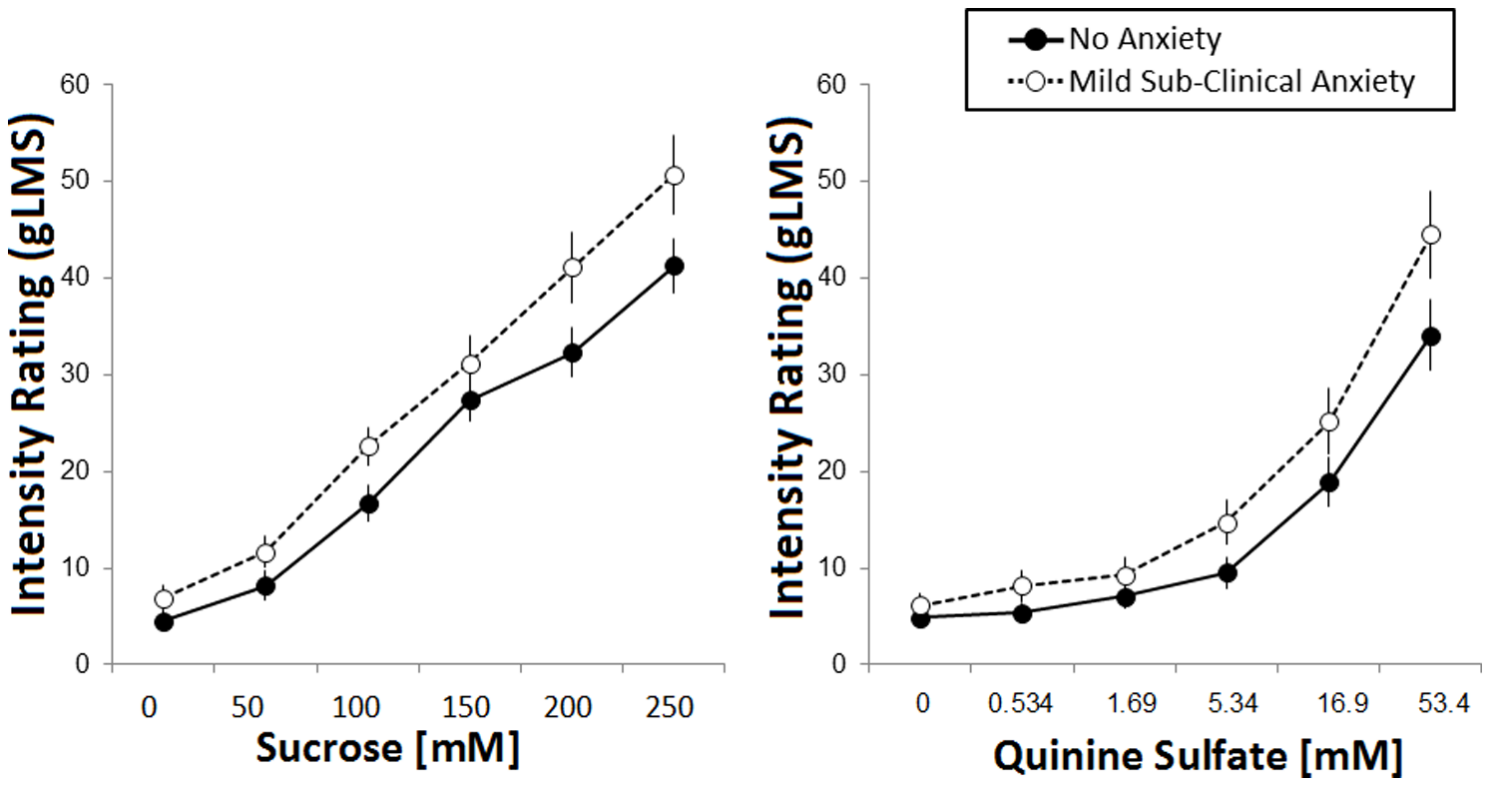
Mean intensity ratings (gLMS) of sucrose (left panel) and quinine sulfate (right panel) during baseline in subjects with no anxiety (filled circles) and mild sub-clinical anxiety (open circles). Error bars are standard errors.

Baseline measurements revealed significant group differences in taste ratings of sucrose (F (1,79) = 5.78, p = 0.018) and quinine (F (1,79) = 5.37, p = 0.023) with the mild subclinical anxiety group providing higher ratings than the no anxiety group (Figure 4). No significant group differences were found for citric acid, glutamate and fat ratings (all p>0.1). No significant differences in taste ratings were found between the groups with mild subclinical anxiety exhibiting either no or mild subclinical depression (all p>0.01).

#### Interaction of “subclinical” trait-anxiety and mood induction

There were no specific interaction effects of mood induction and the anxious subject groups on taste ratings: sucrose: F(10,70) = 0.503; p = ns; quinine: F(10,70) = 1.254; p = ns; citric acid: F(10,70) = 0.610; p = ns; umami: F(10,70) = 0.744; p = ns; fat: F(10,70) = 1.307; p = ns).

## Discussion

The present study investigated whether oral perceptions of fat and taste stimuli are modulated by affective state, anxiety, and/or by experimental manipulations of mood. To our knowledge this is the first study to investigate both the effects of mood and affect on the perceptions of taste stimuli (quinine sulfate, sucrose, citric acid, and monopotassium glutamate) and dairy fat. Regarding fat, recent evidence suggests that consumption of fat may play a major role in eating disorders, especially affecting emotional eating while in negative mood [32]. The hypothesis of an effect of mood induction on fat perception may explain the associations among obesity, high negative affect, and overeating under negative emotions as reported by Jansen et al. [33].

In this study, we observed that among the oral sensory stimuli examined, the ratings of fat were indiscriminant of fat concentration in the mildly subclinical depressed group, but only after the induction of positive or negative mood. That is, people with mild subclinical depression were not able to rate fat intensities according to concentrations after either the positive or the negative mood induction. Laeger et al [34] found correlations between subclinical anxiety and depression and amygdala responses to negative words. The range of BDI and STAI-trait scores of their clinically healthy subjects was comparable to the range our subjects. At present, we do not know how oral fat perception was affected by mood and affective state, and we do not know why only fat but not taste perceptions were affected this way. A possible implication, however, is that these subjects might unwittingly ingest greater amounts of fat as a result of their apparent inability to perceive differences in fat concentrations under conditions of elevated mood.

Whereas a decrease in the ability of slightly subclinically depressed subjects to rate fat concentrations accurately when in negative or positive mood has not previously been reported, others have shown that obese people underestimate their energy and fat intake [35]–[37]. In the present sample, the correlation between depression score (BDI) and body mass (BMI) was *r* = 0.400 (p = 0.007) for the subjects in the group with the sub-clinical mild depression. No significant correlation for the two variables was found in the non-depressed group (*r* = −0.161 (p = ns)). Interestingly, Stewart et al. [38] found a higher BMI in people with a low sensitivity to fat among presumably clinically non-depressed subjects. However, in the absence of any mood manipulation, we did not see a difference in ratings of fat content between the groups with high and low affective traits. A general association between sad and happy mood induction by video clips and increased ratings of taste was found for citric acid. This effect is consistent with observations that emotions (both positive and negative) can augment intensity in taste [39] or in smell [40], presumably via mechanisms of emotional arousal. Indeed both films were rated as significantly higher in arousal than the neutral movie. The enhanced state of arousal might have heightened the response to the sour stimuli, although the same effect did not occur for the other taste stimuli.

Within the range of sub-clinical depression and - anxiety state variations of subjects, increased depression and anxiety indices (which were correlated r = 0.69 with each other) were associated with increased taste intensity ratings of sucrose, quinine, and to a slightly lesser degree citric acid. Therefore, there was an overall tendency for elevated mood, both positive and negative, and elevated subclinical depression and anxiety to predict higher taste intensity ratings of quinine, sucrose and citric acid.

We believe that our data on sweet, sour and bitter tastes support prior observations of a relationship between subclinical depression and anxiety and taste thresholds, although previous work on this topic has focused on threshold measures. Healthy individuals who are more anxious are demonstrably more sensitive to sensory inputs. Anxious people are more sensitive to pain [41], to tone loudness [39],to threatening faces [42], to unpleasant odorants [43] and to bitter [44] or salty taste [14]. Non-clinical subjects with mild anxiety are also more sensitive to threatening information, which is explained by a generalized enhanced vigilance in this subject group [45]. In our study there were no significant differences between mildly subclinical depressed subjects with no and mild subclinical anxiety. Similarly, there were no differences between mildly subclinical anxious subjects with no and mild subclinical depression. Thus, both subclinical depression and - anxiety traits did not appear to be affecting ratings independently.

Other studies of taste intensity ratings in non-clinical populations have had more discrepant results: A main effect of stress induction on taste ratings was found by Dess and Edelheit [39]. In the stress condition subjects rated saccharin’s bitterness higher than in the control condition. Dess and Chapman [46] also tested the relationship between depression and bitter taste in a nonclinical sample. They found an association between higher BDI scores and higher taste ratings generally. In contrast, no correlations between depressive symptoms in a non-clinical sample and taste intensity ratings for sour and bitter were found in the study by Scinska et al., [47]. Negative correlations have also been reported between subclinical depression and the intensity of sweet taste ratings by Al’Absi [48] and Scinska et al., [47]. Studies of clinical populations revealed divergent results as well. From the theory of anhedonia in major depression disorder (MDD), we would expect lower intensity ratings for sweet taste in patients with major depression compared to healthy control groups, a finding that was observed by both Berlin et al. [49] and Amsterdam et al. [16]. But no difference in the sensitivity to sucrose between patients with MDD and a control group was found by Dichter et al. [50].Thus, the present findings of elevated taste ratings in subjects with subclinical depression and during mood manipulations are consistent with the observations of some prior reports, but not others. Possible explanations for differences among studies could be different psychophysical techniques for taste evaluations and different levels of mood alteration, which could be associated with the induction of different neuropharmacological states.

None of our manipulations were associated with significant variation in the taste of the amino acid glutamate. There were no significant differences in the intensity ratings of glutamate as a function of mood manipulation or affective traits. But glutamate has atypical taste properties compared to other traditional taste compounds. Beauchamp [51] stated that the savory perception of glutamate is different from salt, sweet, bitter, and sour, in that it is has more mouthfeel characteristics. It is also undesirable by itself, unlike the tastes of sugar or dilute acid or salt, and requires combination with other tastes and flavors, especially salty, to be desirable.

Heath et al [7] demonstrated that the pharmacological manipulation in non-clinical subjects of 5HT and noradrenergic neurotransmitter systems is associated with changes in taste perception. These neurotransmitters have independently been demonstrated to be important to taste signaling. We suggest that non-clinical variations in these neurotransmitter systems manipulated by changes in mood and affect were responsible for the altered taste and fat perception observed in the present report.
